# Round the Hospitals

**Published:** 1921-09-10

**Authors:** 


### ROUND THE HOSPITALS.

The Rules of the General Nursing Council for Ireland, which are now to hand as finally approved, have been already discussed in these columns. They contain the conditions of admission of existing nurses and of interim nurses to the General Part of the Register and to the Supplementary Parts for male nurses, mental nurses, sick children's nurses, and fever nurses. Existing nurses must have (*a*) not less than three years' approved training in a general hospital or Poor-Law infirmary, or (*b*) not less than one year's such training with two years' subsequent *bona-fide* practice, or (*c*) been employed with the approval of the Local Government Board for Ireland for at least three years as a "qualified" nurse within the meaning of the Board's General Order of July 5, 1901. This last qualification is quite the lowest laid down for admission to the General Register under the three General Nursing Councils. It is not probable, however, that any large number of "qualified" nurses will seek admission to the ranks of private nursing through the Register. "Interim" nurses must have had three years' satisfactory training in a general hospital or Poor-Law infirmary approved by the Council, and the rules for the training of existing candidates for admission to the Supplementary Parts of the Register conform to the standard in England and Scotland.

The London County Council have issued fresh by-laws for the regulation of licensed establishments for massage or especial treatment. The principal innovation is that a complete scale of fees or charges for massage or special treatment shall be placed in conspicuous positions, so that they can be seen by

**ROUND THE HOSPITALS**—(*continued*).

all who receive treatment. No variation is permitted from the public scale of fees. Stringent rules about advertising are now to be enforced. No licensed person may advertise that either he or his establishment is licensed by the Council, nor may advertise at all in any public thoroughfare or convenience or conveyance. Advertisement is only permitted in or on the premises or in newspapers publicly exposed for sale. Other regulations prohibit the permission of any indecent or disorderly act, the employment or admission of persons of known immoral character, and the locking of doors of patients under treatment. The records which must be kept of all cases are to be open to the inspectors.

---

The attention of housekeepers should be drawn to a passage in the Report of the Ministry of Health dealing with egg powders. It is customary for these to be advertised as perfect substitutes, imparting the lightness, richness, flavour, and appearance of new-laid eggs. It is pointed out that egg powders are simply coloured baking powders; that the samples submitted contained only 0.3 to 0.8 per cent. of fat, while eggs contain about 10 per cent. of fat, or twenty times as much as in the powders; while the protein contents are 14 per cent. in eggs, as against about 5 per cent. belonging to the rice flour in the egg powder. The price charged for the powder varies from 1d. to 2d. per oz., and we are warned that there is no relation between the cost and the value. A good deal of adulteration has been taking place in the manufacture of vinegar, including the use of artificial vinegar, coloured and dilute acetic acid, and the addition of water in large proportions.

---

The Metropolitan Asylums Board has it in contemplation to make arrangements for the training of the kitchen staff, which numbers 300 women, in the various institutions under its control. Many of these employees are unskilled, and there is every reason to believe they could be rendered more efficient by a system of regular training and promotion. The comfort of patients and staff alike is intimately concerning in raising the standard of efficiency in the kitchen, and it cannot be doubted that economy is promoted by sound training. The Board is notable for its fine kitchen equipment and the provision of labour-saving devices. The progress of the new scheme of training will be watched with interest, and we trust it may meet with imitation.

---

The tennis grounds of the St. Marylebone Infirmary were the scene of an animated contest on the last day of August, when the final round of the Challenge Cup Competition for the nurses of London was played. The London Hospital nurses, holders of the cup, beat those of Guy's Hospital by two matches to love.

---

The foundation-stone of a new electro-massage department was laid on August 22 at the Royal Infirmary, Preston, in the presence of a large assembly, and on the same occasion training-school badges and prizes were distributed to the recently qualified nurses. Miss Elsia Lang obtained the highest number of marks in the final examination, and is to receive free midwifery training as a scholarship. Dr. Collinson, in the course of an address on the training of nurses, declared the three motive forces in its development were religion, science, and war. He suggested that Government aid to the hospitals might take the form of grants towards the training of the resident medical staff and towards the teaching of nursing by a special sister tutor. The new East Ward, which was opened by the Mayoress, contains seventeen beds, and is to be reserved for the use of Service men needing recurring treatment.

---

That excellent periodical the New York *Trained Nurse and Hospital Review* has sustained a great loss in the retirement of Mrs. Annette Sumner Rose, publisher and directing editor for thirty-three years. The new management promise more illustrations and a continuance of the progressive nursing standard which has set this journal very high among its contemporaries.

---

The Eastbourne Guardians have arranged to give a clear day off duty every week to their nurses. The duty hours of the day staff have been reduced to a daily average of nine and a-quarter, and the night hours to eleven, or a weekly average of fifty-five and a-half and sixty-six. Holidays are to be: superintendent nurse, twenty-eight days; sisters and staff nurses, twenty-four; probationers, sixteen. The night hours are too long, and arrangements should be made by a system of relief to give the nurses a period of two hours off duty during the night, with a meal outside the wards.

---

The Manchester Children's Hospital at Pendlebury has always been successful in attracting a large circle of sympathisers to co-operate in the labours of the permanent staff. The Ladies' Auxiliary Fund is particularly well organised, and this fact was brought into prominence at its recent annual meeting. The report states that there are now thirty-one districts included in the operations of of the Fund, of which twenty-nine have each subscribed £50 or more in support of a cot for the year. In all fifty-six cots are maintained by the Fund, many of the districts supporting more than one. The amounts collected totalled £3,258, besides £6,493 towards the "Special Effort" Fund to free the hospital from debt.

---

The Salford Guardians are faced with the necessity for undertaking great improvements at the Hope Hospital, involving the erection of a new laundry, a new central heating instalment, and the enlargement of the Nurses' Home. Much dissatisfaction has been caused by the decision to defer the latter much-needed extension. More nurses are being engaged, yet there is already not sufficient room for the present staff. One of the lady Guardians made a formal protest, and is on strong ground in her warning that the Guardians cannot expect good nursing unless the nurses receive proper treatment.

The movement for training Indian women to be nurses, and providing them with hostels, started at Calcutta three years ago, and at a reception given by Lady Mookerjee, at 21 Cromwell Road, S.W. 1, to meet the Committee of the Indian Nurses' Hostel Fund, very satisfactory progress was reported by the Secretary, Lady Rogers. Sir Henry Ledgard, in sending a donation of 1,000 rupees, intimated that he intended to build a hostel at Cawnpore for the training of twenty girls. The housing difficulty is even more acute than in this country, and must be overcome first.

The hospital committee of the Wandsworth Guardians recently reported the resignation of a probationer who had previously made a complaint as to her treatment by her ward sister. A thorough investigation into the circumstances was made, the officers concerned were interviewed, and the committee recommended that in the interests of the institution the resignation be accepted, although it was reported that the probationer desired to withdraw it. She had in effect, it would seem, effectually called attention by her complaint of her superior officer to the fact that her record was unsatisfactory in nearly every ward she had worked in, and the decision of the committee that she was unsuitable for further training was arrived at by a large majority. Miss Hill, in an able speech, likened the hospital to an army fighting disease and death, of which the officers were the staff. They could never wage such a fight successfully with an undisciplined army. The recommendation of the committee was adopted.

Nursing in the Isles of the West is carried on under unusually difficult conditions. The population is scattered, and in spite of the Board of Health grant of 50 per cent. of the approved expenditure for the year, it is hard to get together funds sufficient to maintain the nurse. At North Uist the Nursing Association has adopted a contributory basis, each cottar to pay 2s. 6d. a year, and tenant farmers, feuars, merchants, and landholders 7s. 6d. The original scheme approved by the Medical Service Board estimated for four nurses for the island, but at present funds barely suffice for two, and it is hardly possible that all cases can receive attention.

A notable victory has been won over the nursing arrangements at the Kendal Workhouse. Under former arrangements the nurses, as the Chairman expressed it, had been at the command of the sick day and night, which means probably that there was little if any night nursing. It has now been decided to increase the staff for the sick wards, containing thirty occupied beds, to four, and thus the sick will have the attention they require night and day.

The *Sphere* of September 3 has a finely illustrated article on St. Bartholomew's, which should not be missed by any who are interested in "London's Oldest Hospital." A group of the ward sisters with Miss McIntosh is excellent, and the "Nurses at Lunch" is also effective. Two scenes from the Elizabeth Maternity Ward have also come out well; there are many good portraits.

